# Remembering Prof. Il-Woo Nam: a pioneering surgeon and a compassionate mentor

**DOI:** 10.1186/s40902-023-00407-4

**Published:** 2023-10-26

**Authors:** Seong-Gon Kim

**Affiliations:** Department of Oral and Maxillofacial Surgery, College of Dentistry, Gangneung-Wonju National University, Jibyun-Dong, Gangneung, Gangwondo 28644 Republic of Korea

**Fig. 1:**
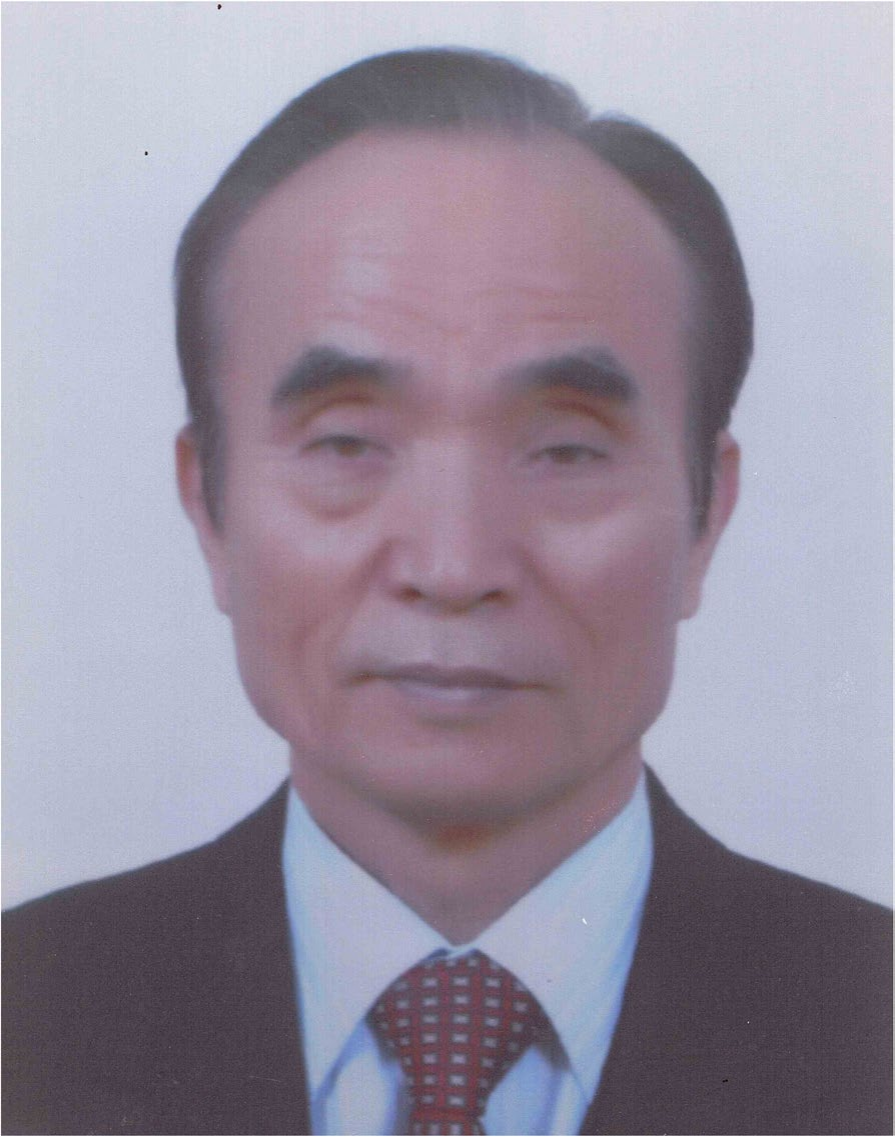
Portrait of Prof. Il-Woo Nam

It is with profound sorrow that we bid farewell to Prof. Il-Woo Nam (Fig. 1), who tragically left us on September 19, 2023, following a car accident. As per Korean tradition, Prof. Nam and his beloved wife were returning from a visit to their parents’ grave on Korean Thanksgiving Day, a day marked by paying homage to ancestors, when the unforeseen mishap took place, claiming both their lives.

Prof. Nam was born in Cheonan, Republic of Korea, and was a proud alumnus of Seoul National University where he later pursued his specialization in oral and maxillofacial surgery at the Seoul National University Hospital. His academic journey came full circle when he took up the mantle of a professor at Seoul National University, a position he held with distinction from 1967 to 1998.

In his illustrious career spanning over three decades, Prof. Nam made groundbreaking contributions to the surgical realms of maxillofacial trauma, cleft lip and palate surgery, and various other jaw and facial surgeries. His tenure as the President of the Korean Association of Oral and Maxillofacial Surgery from 1992 to 1994 was marked by substantial advancements in the field.

Those fortunate enough to work alongside Prof. Nam, including myself during my residency, were privy to his humble and modest demeanor. He invariably prayed before commencing any surgical procedure, entrusting the well-being of his patients to a higher power. He was a firm believer in collaborative effort, attributing the success of his surgeries not solely to his exemplary skills but to the aid and expertise of his colleagues.

Upon his retirement, Prof. Nam’s insatiable desire to heal led him abroad where he performed surgeries for cleft lip and palate in China and Egypt in 2001 and 2003, respectively. His altruistic endeavors were a testament to his unwavering dedication to his profession and humanity.

**Fig. 2:**
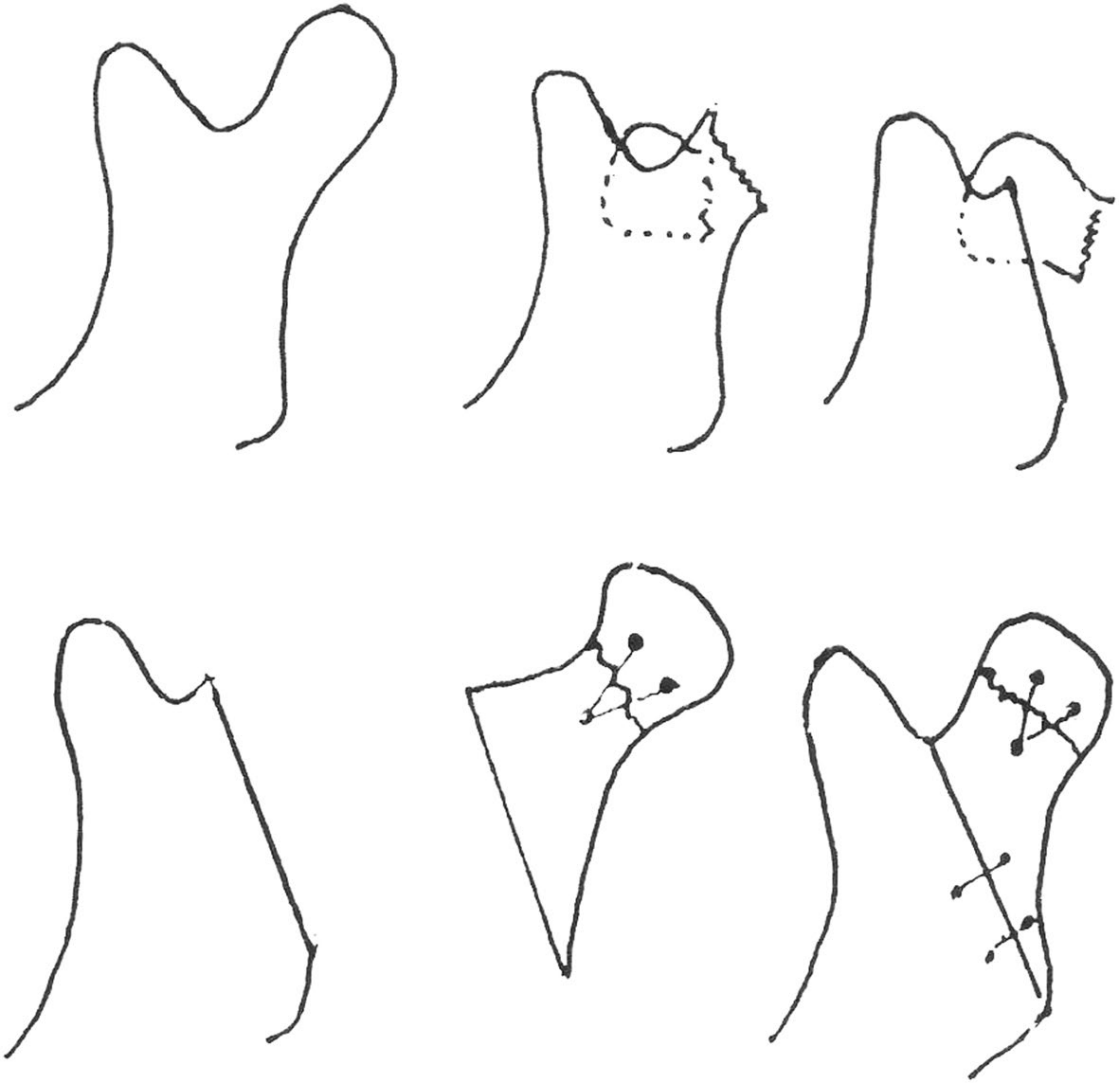
Illustration depicting Prof. Nam’s surgical technique

Prof. Nam’s legacy in the medical field is immortalized by the “Nam's Method”—a novel approach to mandibular condyle surgery, which entailed the extracorporeal reduction of mandibular condyle fractures (Fig. 2). This method, first published in 1978, showcased a highly innovative idea of extracorporealization of the condylar segment to facilitate open treatment of condylar fractures [1]. This became a viable treatment option when conventional approaches fell short. His initial publication documented 25 successful cases treated with this method, and by 1980, the successful surgical outcomes were widely recognized and published [2]. The essence of the “Nam's Method” continues to resonate in recent publications [3], underscoring its relevance and significance in contemporary surgical practices.

As we mourn the loss of Prof. Il-Woo Nam, we also celebrate a life replete with professional excellence, selfless service, and a nurturing mentorship that has left an indelible mark on countless lives. His memory will continue to inspire and guide us in our endeavors, embodying the epitome of medical altruism and educational stewardship.
